# Trends in dispensing errors reported in Finnish community pharmacies in 2015–2020: a national retrospective register-based study

**DOI:** 10.1186/s12875-024-02428-y

**Published:** 2024-05-23

**Authors:** Emilia Mäkinen, Anna-Riia Holmström, Marja Airaksinen, Anna Schoultz

**Affiliations:** 1 Clinical Pharmacy Group, Division of Pharmacology and Pharmacotherapy, Faculty of Pharmacy, University of Helsinki, Helsinki, Finland; 2 Pharmacy Kaari, Helsinki, Finland

**Keywords:** Community pharmacy, Dispensing error, Medication error, Medication error reporting, Medication safety, Medicines verification system, Outpatient care, Patient safety, Primary care

## Abstract

**Background:**

Community pharmacies are responsible for dispensing of medicines and related counselling in outpatient care. Dispensing practices have remarkably changed over time, but little is known about how the changes have influenced medication safety. This national study investigated trends in dispensing errors (DEs) related to prescribed medicines, which were reported in Finnish community pharmacies within a 6-year period.

**Methods:**

This national retrospective register study included all DEs reported to a nationally coordinated voluntary DE reporting system by Finnish community pharmacies during 2015–2020. DE rates, DE types, prescription types, individuals who detected DEs and contributing factors to DEs were quantified as frequencies and percentages. Poisson regression was used to assess the statistical significance of the changes in annual DE rates by type.

**Results:**

During the study period, altogether 19 550 DEs were reported, and the annual number of error reports showed a decreasing trend (*n* = 3 913 in 2015 vs. *n* = 2 117 in 2020, RR 0.54, *p* < 0.001). The greatest decrease in reported DEs occurred in 2019 after the national implementation of the Medicines Verification System (MVS) and the additional safety feature integrated into the MVS process. The most common error type was wrong dispensed strength (50% of all DEs), followed by wrong quantity or pack size (13%). The annual number of almost all DE types decreased, of which wrong strength errors decreased the most (*n* = 2121 in 2015 vs. *n* = 926 in 2020). Throughout the study period, DEs were most commonly detected by patients (50% of all DEs) and pharmacy personnel (30%). The most reported contributing factors were factors related to employees (36% of all DEs), similar packaging (26%) and similar names (21%) of medicinal products.

**Conclusions:**

An overall decreasing trend was identified in the reported DEs and almost all DE types. These changes seem to be associated with digitalisation and new technologies implemented in the dispensing process in Finnish community pharmacies, particularly, the implementation of the MVS and the safety feature integrated into the MVS process. The role of patients and pharmacy personnel in detecting DEs has remained central regardless of changes in dispensing practices.

**Supplementary Information:**

The online version contains supplementary material available at 10.1186/s12875-024-02428-y.

## Background

Community pharmacies have a core position in ensuring the safe use of medicines in outpatient care [1–8]. Their main responsibilities comprise medication dispensing and related counselling [9]. Community pharmacists ensure that the right medication with the right dose and administration route will be dispensed to the right patient during the dispensing process. The process also involves double-checking the appropriateness of the prescription (e.g. dosages are within recommended dose limits), identifying possible duplicate prescriptions and potentially harmful drug-drug interactions, as well as supporting patient’s adherence and self-management of the treatment.

Even though community pharmacists dispense medications according to the national guidelines set the minimum standards for pharmacy practice [9, 10], human errors may occur. An unintentional deviation from the prescription while dispensing in the community pharmacy is called a dispensing error (DE) [11]. A DE may occur, e.g. if a pharmacist mistakenly selects the wrong strength of the medicine while dispensing. In previous studies, dispensing the wrong strength of medicine, the wrong medicine and the wrong quantity of medicine have been identified as the most common types of DEs [12–22]; the incidence of DEs have varied between < 1% and 55% depending on the dispensing system, operational definitions and research method used in the study [12, 19–21, 23–25].

In addition to DE types and incidence, previous studies have investigated clinical significance, causes, contributing factors and predictors of DEs and their prevention strategies with different methods [6, 7, 12–36]. However, most of the earlier studies were carried out by cross-sectional designs and they have often been geographically limited, e.g. comprising only a single city [7]. None of the studies has focussed on investigating national trends in DEs, although it could reflect the development of the dispensing process safety and functioning of implemented defences. Consequently, this national study aims to investigate trends in DEs related to prescribed medicines, which were reported in Finnish community pharmacies within a 6-year period of 2015–2020.

## Methods

### Study context

Finland, with a population of 5.6 million inhabitants, has a private community pharmacy system with over 800 outlets owned by pharmacy professionals [9]. The Finnish Medicines Agency Fimea regulates the community pharmacy system and the number of pharmacy outlets to ensure commitment to the national health policy goals [8, 9, 37]. Medicines (prescription and non-prescription) are distributed to outpatients by community pharmacies. Only licenced pharmacists are allowed to dispense medicines and provide related counselling. Most of the prescriptions are valid for two years’ supply, and due to the reimbursement rules of the Social Insurance Institution of Finland (Kela), prescription medicines are dispensed to patients in amounts equivalent to a maximum of three months’ use at a time. In Finland, medications are dispensed to patients in their original packages prepacked by the manufacturers according to the harmonised EU regulation on packaging and labelling [38–40].

#### Evolution of the dispensing process

The dispensing process has remarkably changed in Finnish community pharmacies over time [41]. Prescriptions have been electronically processed in all Finnish community pharmacies since the 1980s. The first electronic medicines information system for prescription medicines was also launched in the 1980s, later followed by more sophisticated systems assisting community pharmacists in counselling and identifying medication-related risks, such as drug-drug interactions [41–43]. These systems have evolved into comprehensive medication risk management systems integrated into the dispensing process, and the same national electronic databases are being used by physicians and other healthcare providers in their practices [41–45].

The most recent major technological advancements in the dispensing process have been the national introduction of electronic prescription in 2017 (after a transition period since 2011) and the launch of the Medicines Verification System (MVS) maintained by Finnish Medicines Verification (FiMVO) in 2019 [46–48]. The MVS has become mandatory within European Union countries to prevent falsified medications from entering the legal supply chain by ensuring safety features in the medication packages [46, 49, 50]. With the introduction of the MVS, each medication package was included a 2D data matrix code, and their scanning became a mandatory part of the routine dispensing process. In Finland, an additional safety feature was added to all community pharmacies’ electronic prescription processing systems and integrated into the MVS process; while dispensing, when the 2D code of the medication package is scanned due to the MVS, at the same time, the added safety feature compares the information of the collected medicinal product (using 2D code information) to the product chosen from the electronic prescription processing system of the community pharmacy. Consequently, the safety feature integrated into the MVS process confirms that the right product is collected from medicine storage and dispensed to the patient. When the information between the medication package’s 2D code and the product chosen from the electronic prescription processing system does not match, the system generates an alert. Thus, the defence integrated into the MVS process adds to the safety features of dispensing robotics, which has been widely used in large-volume Finnish community pharmacies since the late 2000s.

#### DE reporting in Finnish community pharmacies

Finnish community pharmacies have been obliged to document and manage DEs since 2012 [10]. However, according to a national study, most community pharmacies had already established their own practices to document and manage DEs by 2005 [29, 30]. The DE reporting and management became systematic and nationally coordinated in 2012 when the Association of Finnish Pharmacies (AFP) established the national electronic DE register for their member community pharmacies. The AFP is a professional organisation of community pharmacy owners, covering the majority of community pharmacies in Finland [51]. During the study years 2015–2020, 95–97% (*n* = 773–792) of Finnish community pharmacies were AFP members.

The use of the AFP’s electronic DE reporting system has been voluntary, as DEs can also be documented manually. The DE reporting system is designed to support systems-based and blame-free DE reporting, providing community pharmacies with retrospective information on the encountered safety risks associated with their dispensing process [52–54]. The DE reporting system records the data in a structured and open narrative form [Additional file 1]. The applied DE taxonomy system is adopted from the American Society of Consultant Pharmacists [Additional file 2]. The aggregated DE report data entered into the national AFP register have been previously analysed only once in 2014, covering a 13-month period in 2013–2014 [26].

### Study design and method

This was a national retrospective register study including DEs reported to the voluntary national DE register maintained by the AFP during a 6-year period from January 2015 to December 2020. All member community pharmacies of the AFP had the opportunity to participate in this study using the DE reporting system. This study applied the human error theory as a theoretical framework [55]. The theory is widely used as an approach to improve the safety of health- and social care systems. Complex health- and social care systems are error-prone environments in which, according to the theory, human errors are inevitable, and effective and continuous safety development requires system-oriented thinking. In this study, the theory manifested as a systems approach to DEs and medication risk management.

### Data processing

The data for this study were received from the AFP as a register owner in Microsoft Excel format in February 2022. Information on the DE type, prescription type, individual who detected the DE and contributing factors were derived from the DE reports for each DE included in this study [Additional file 1].

**Fig. 1:**
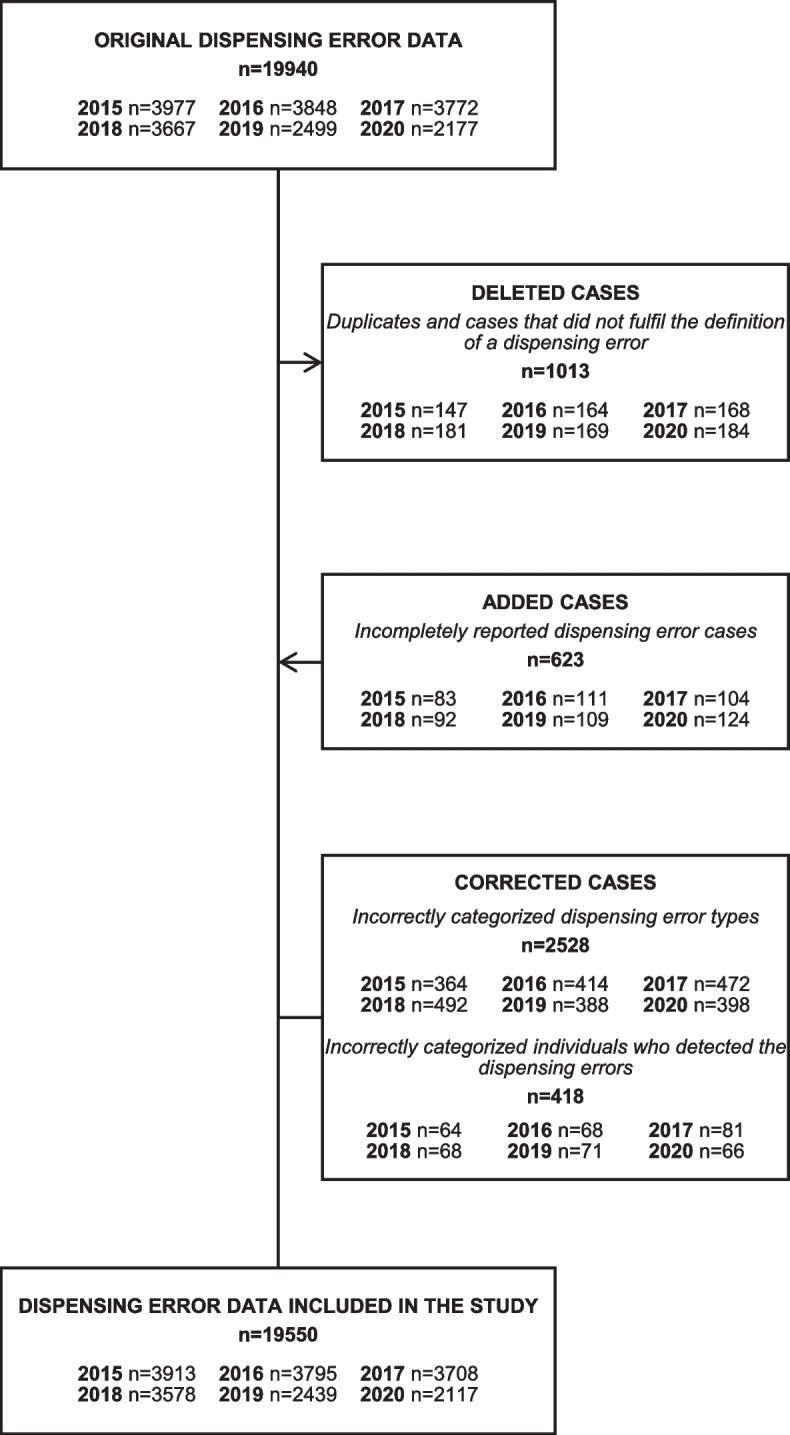
Compiling the research data (*n* = 19 550) from the national dispensing error register data (*n* = 19 940)

 For the statistical analyses, the original data were manually reviewed for technical mistakes and deficiencies in incident documentation and corrected by two researchers (EM, AS) in cases where obvious mistakes or deficiencies occurred in the reports [Fig. 1 and Additional file 3]. This review comprised correcting incorrectly categorised DE types (*n* = 2 528) and incorrectly categorised individuals who detected the DEs (*n* = 418), deleting duplicates and reports not fulfilling the definition of a DE (*n* = 1 013). Such cases included errors, which were detected and corrected before the medicine was dispensed from a community pharmacy, prescribing errors and other errors that occurred elsewhere in the health- and social care system, errors in dispensing over-the-counter medicines, errors related to processing direct compensations of medicines granted by the Social Insurance Institution of Finland (Kela) and deliberate deviations from prescriptions made due to pharmacists’ consideration.

Researchers also reviewed and added incompletely reported DEs (*n* = 623) to the research data [Fig. 1 and Additional file 3]. The incompletely reported cases were comprised of reports where the errors were recorded as one error, although the case included various types of independent DEs (e.g. the wrong strength and pack size were dispensed to the patient). These cases were recorded as separate DEs for the analysis, as well as cases in which the reported DE was related to more than one medicinal product. Error cases that had repeated more than once per patient were treated as individual cases according to the number of their recurrence.

### Data analysis

A descriptive quantitative analysis of the data was performed using Microsoft Excel. The data was analysed annually and by combining all the annual data from 2015 to 2020. DE types, prescription types, individuals who detected DEs and contributing factors to DEs were presented as frequencies and percentages.

Poisson regression was used to analyse changes in the annual absolute and relative numbers of DE types. Changes in the annual absolute and relative numbers of different DE types were compared (1) between the first and the last year of the study period (2015 vs. 2020) and (2) before and after the introduction of the MVS and the safety feature integrated into the MVS process (2015 vs. 2018 and 2018 vs. 2020). Due to the transitional period, not all medication packages had 2D codes enabling the use of the MVS by the beginning of 2019, 2020 was used for the statistical comparison instead of 2019. The relative risk (RR) of annual changes in absolute and relative numbers of DE types was calculated. The change was considered statistically significant when the p-value was less than 0.05. The statistical analyses were performed by IBM SPSS Statistics 29 for Windows (IBM Corp., Armonk, NY).

## Results

Depending on the year, 59–68% of the AFP member community pharmacies had reported DEs to the national DE register during the study period. The original DE data reported in 2015–2020 included 19 940 DEs [Fig. 1]. After data processing, the final research data comprised 19 550 DEs.

**Fig. 2:**
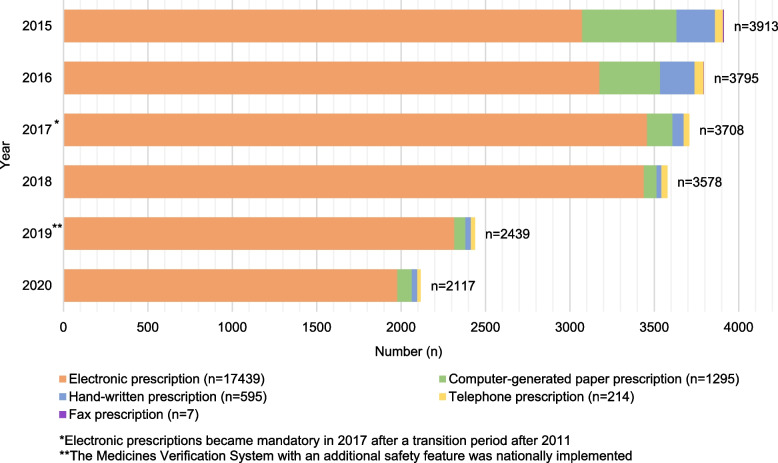
The decreasing trend and annual distribution (n) of reported dispensing errors (*n* = 19 550) by prescription types

 During the study period, the annual number of all DE reports decreased significantly from 3 913 reports in 2015 to 2 117 reports in 2020 (RR 0.54, *p* < 0.001) [Table 1; Fig. 2]. The decrease was deepest during the last years of the study period (2018 vs. 2020, RR 0.59, *p* < 0.001).

### DE types

The annual number of DE types decreased significantly during the study period, apart from the errors related to multidose dispensing (*n* = 121 in 2015 vs. *n* = 188 in 2020, RR 1.55, *p* < 0.001) and preparing medication for administration (*n* = 66 vs. *n* = 144, RR 2.18, *p* < 0.001) that increased significantly [Table 1]. Only the annual number of errors related to the wrong person or name did not change statistically significantly during the study period. The most common DE type was the wrong strength of the medicine dispensed, comprising 50% of all DEs (*n* = 9 849/19 550 in 2015–2020). The most remarkable decrease in the percentage units of errors was achieved in wrong strength errors in dispensing (54% of all DEs in 2015 vs. 44% in 2020).

**Table 1:** Annual dispensing error rates and types reported by community pharmacies during 2015–2020

Dispensing error type	2015	2016	2017	2018	2019	2020	TOTAL	2020 vs. 2015*		2018 vs. 2015*		2020 vs. 2018*	
	*n* *%*	*n* *%*	*n* *%*	*n* *%*	*n* *%*	*n* *%*	*n* *%*	*RR*	*p*	*RR*	*p*	*RR*	*p*
Wrong strength	2121	1979	1913	1814	1096	926	9849	0.44	< 0.001	0.86	< 0.001	0.51	< 0.001
	54.2	52.1	51.6	50.7	44.9	43.7	50.4	0.81	< 0.001	0.94	0.037	0.86	< 0.001
Wrong quantity or pack size	545	536	526	517	273	115	2512	0.21	< 0.001	0.95	NS	0.22	< 0.001
	13.9	14.1	14.2	14.4	11.2	5.4	12.8	0.39	< 0.001	1.04	NS	0.38	< 0.001
Wrong medicine	249	251	259	261	171	160	1351	0.64	< 0.001	1.05	NS	0.61	< 0.001
	6.4	6.6	7.0	7.3	7.0	7.6	6.9	1.19	NS	1.15	NS	1.04	NS
Wrong dosage form	201	219	206	181	155	146	1108	0.73	0.003	0.90	NS	0.81	NS
	5.1	5.8	5.6	5.1	6.4	6.9	5.7	1.34	0.007	0.98	NS	1.36	0.005
Error related to multidose dispensing	121	89	109	125	153	188	785	1.55	< 0.001	1.03	NS	1.50	< 0.001
	3.1	2.3	2.9	3.5	6.3	8.9	4.0	2.87	< 0.001	1.13	NS	2.54	< 0.001
Error in preparing medication for administration	66	88	105	90	129	144	622	2.18	< 0.001	1.36	NS	1.60	< 0.001
	1.7	2.3	2.8	2.5	5.3	6.8	3.2	4.03	< 0.001	1.49	0.014	2.70	< 0.001
Pricing error	101	130	116	114	70	47	578	0.47	< 0.001	1.13	NS	0.41	< 0.001
	2.6	3.4	3.1	3.2	2.9	2.2	3.0	0.86	NS	1.23	NS	0.70	0.037
Wrong person or name	97	106	73	71	67	87	501	0.90	NS	0.73	0.046	1.23	NS
	2.5	2.8	2.0	2.0	2.7	4.1	2.6	1.66	< 0.001	0.80	NS	2.07	< 0.001
Incorrectly recorded dosage instructions at the pharmacy	99	71	46	41	33	33	323	0.33	< 0.001	0.41	< 0.001	0.80	NS
	2.5	1.9	1.2	1.1	1.4	1.6	1.7	0.62	0.016	0.45	< 0.001	1.36	NS
Wrong generic medicine	39	42	42	45	24	20	212	0.51	0.015	1.15	NS	0.44	0.003
	1.0	1.1	1.1	1.3	1.0	0.9	1.1	0.95	NS	1.26	NS	0.75	NS
Dispensing entries made incorrectly by the pharmacy	29	43	41	36	20	15	184	0.52	0.038	1.24	NS	0.42	0.004
	0.7	1.1	1.1	1.0	0.8	0.7	0.9	0.96	NS	1.36	NS	0.70	NS
Other	245	241	272	283	248	236	1525	0.96	NS	1.16	NS	0.83	0.039
	6.3	6.4	7.3	7.9	10.2	11.1	7.8	1.78	< 0.001	1.26	0.007	1.41	< 0.001
All dispensing errors	3913	3795	3708	3578	2439	2117	19550	0.54	< 0.001	0.91	< 0.001	0.59	< 0.001

*Changes in annual absolute (n) and relative numbers (%) of different dispensing error types were analysed using Poisson regression. Changes are compared between 2015 and 2020 and before and after the national introduction of the Medicines Verification System and the safety feature integrated into the medicines verification process (2018 vs 2015 and 2020 vs 2018)

An electronic prescription that became mandatory in Finland in 2017 after a transition period since 2011 was the most common prescription type in the reported DEs (89% in 2015–2020) [Fig. 2]. The proportion of electronic prescriptions in reported DEs by prescription type increased between the years 2015 to 2020 (79% in 2015 vs. 93% in 2020).

### Individuals detecting DEs

**Fig. 3:**
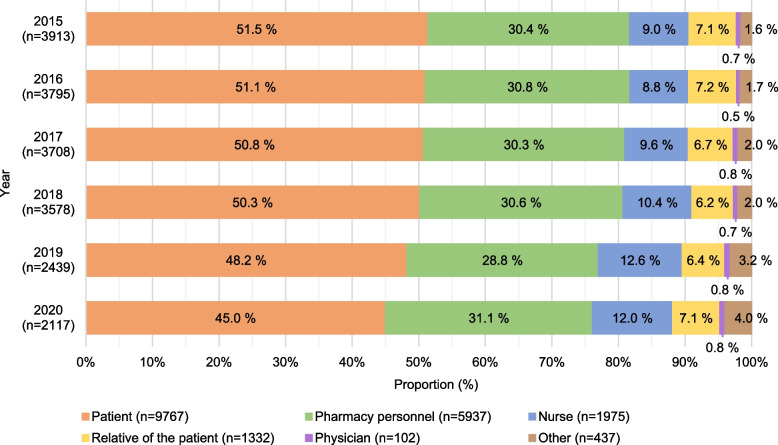
Individuals who detected dispensing errors (*n* = 19 550) (% of the annually reported dispensing errors)

 Throughout the study period, DEs were most commonly detected by patients (50% of the reported DEs) or their relatives (7%) [Fig. 3]. Almost one-third (30%) of the reported DEs were detected by pharmacy personnel. The proportion of patients who detected DEs had slightly decreased from 2015 to 2020 (52% in 2015 vs. 45% in 2020).

### Contributing factors to DEs

**Fig. 4:**
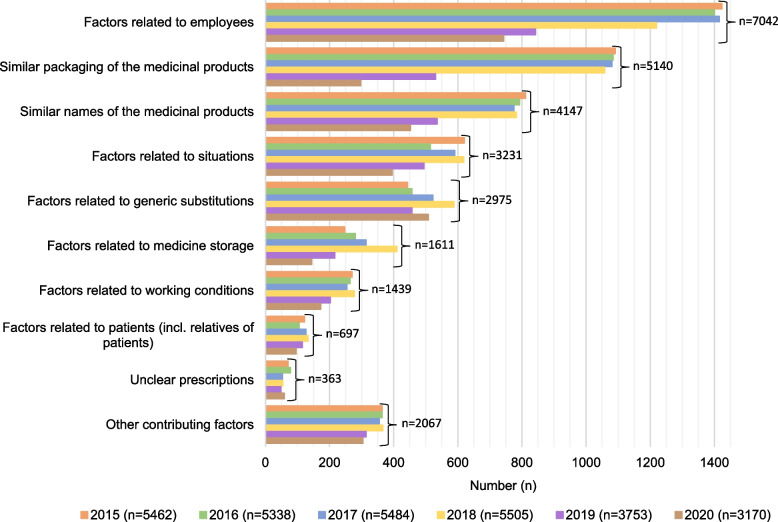
Contributing factors (*n* = 28 712) of the dispensing errors (*n* = 19 550) reported by community pharmacies. Contributing factors had not been defined in 743 dispensing error cases. See Additional file 4 for more detailed statistics on contributing factors

 Community pharmacy personnel reported 28 712 contributing factors to DEs [Fig. 4 and Additional file 4]. At least one contributing factor had been reported for 96% of the DEs (*n* = 18 807/19 550). Throughout the study period, contributing factors related to employees were the most commonly reported (e.g. employee slips of attention, tiredness, or carelessness), and their proportion was 36% of all reported DEs. The proportion of employee-related factors per year remained almost the same (36% in 2015 vs. 35% in 2020). Other common contributing factors were look-alike and sound-alike (i.e. LASA) properties of medicinal products: similar packaging (26% of the reported DEs) and similar names (21%). Examples of such cases were DEs in which pack sizes, strengths, or dosage forms of a medicinal product, generics or additional endings in the names of medicinal products (e.g. comp, plus, forte) were acting as contributing factors. The annual proportion of errors due to similar packaging had decreased (28% in 2015 vs. 14% in 2020), whereas errors contributed by generic substitution increased (11% in 2015 vs. 24% in 2020).

## Discussion

The present study shows that the annual number of DEs reported by Finnish community pharmacies decreased significantly during 2015–2020. The decreasing trend was identified even though the number of prescriptions dispensed by the community pharmacies has grown (55.8 million prescriptions in 2015 vs. 67.1 million in 2020) [56], and the number of community pharmacies using the electronic DE reporting system has maintained constant during the study period. The greatest decrease was observed during the last years (2019–2020) when the MVS and the safety feature integrated into the MVS process was nationally implemented. These findings suggest that nationally implemented systems-based changes in the medication dispensing process in Finnish community pharmacies have most likely greatly impacted dispensing safety. In addition to the safety feature integrated into the MVS process, these systems-based defences arising from the human error theory comprise the national implementation of electronic prescriptions, storage of medicines in the order of sales in pharmacies (i.e. the most commonly dispensed medicines are placed in the most accessible place, and LASA or same-indication medicinal products that can be easily confused with each other are not stored in the same storage place side by side), and dispensing robotics [55]. These all represent preventive risk management actions that reduce human error risk at different dispensing process stages. Although further research is needed to demonstrate the causality of the impact of the actions on dispensing safety, the present study provides preliminary evidence that systematically implemented changes in the community pharmacy practice can improve dispensing process safety.

Almost all types of DEs showed a significant decrease, with the wrong strength being the most common error type. In international studies, DEs related to wrong strength have been among the most common types of DEs, but their proportion (11–31% of all DE types) has been remarkably lower than in this study (50%) [13, 15, 16, 18–22, 28, 36]. That may be due to differences in used error detection methods, definitions of DEs and dispensing processes between countries. Other common errors were dispensing the wrong quantity or pack size and the wrong medicine. The number of all these errors decreased when the MVS and safety feature integrated into the MVS process was implemented in 2019. Therefore, the MVS and process-integrated safety feature have most likely been the major contributor to the descending trend. However, it should be noted that the safety feature can only detect errors occurring in the stage of collecting medication packages from storage while dispensing. If the error has already occurred when a pharmacist is selecting a medicinal product to be dispensed from the electronic prescription processing system, the error will not be detected by the safety feature. This is because the safety feature compares the medicinal product collected from the storage to the product selected from the electronic prescription processing system.

While our findings of the most common types of DEs were similar to previous study findings [12–22], DE trends indicated also some new increasing types of errors; errors related to multidose dispensing and errors in preparing medication for administration (e.g. attaching a wrong dosing instruction label to a medication package). Concerning automated multidose dispensing, the increase in errors is most likely influenced by the increasing use of multidose dispensing services in Finland [57]. These services have become more common in Finland and elsewhere because populations are aging, and the services are mainly targeted to older adults with multiple medications. Future research should be focussed on deepening understanding of the safety risks related to automated multidose dispensing process. This is justified by the fact that errors in multidose dispensing became the second most common error type in the last year of our study (2020). Also, previous studies have raised concerns of the safety risks of the multidose dispensing process [57–59].

The results showed that patients were the main group of individuals detecting DEs, even when the dispensing process had become more electronic due to the implementation of electronic prescriptions and other system-based actions to prospectively manage risks related to manual work and processes. Our findings indicate that the role of patients in ensuring medication safety should be recognised, despite of the technological defences and automation. Therefore, patient involvement in their care is still crucial in medication risk management in the changing health- and social care environments and should be highlighted in future developments, also in community pharmacies. These findings are in line with previous findings and recommendations, even legal requirements for pharmacy practice [1, 7, 60, 61].

The results indicate a decreasing trend in packaging and labelling (LASA) and medicine storage issues as contributing factors to DEs during the last two years of the study period (2019–2020). This decrease may reflect the potential effect of the safety feature integrated into the MVS process on reducing the confusion with the packages of the medicines. Along with risks related to LASA issues with similar packaging (26% of all reported DEs) and names (21%) of the medicines, employee-related issues (36%) were the most reported contributing factors to DEs. Thus, these two types of contributing factors cover the majority (57%) of all reported contributing factors. In the future, more in-depth research into the contributing factors of DEs is needed to identify risk-causing structures and processes, even root causes of errors. Also, strengthening a general understanding of systems-based medication risk management and competence in identifying system weaknesses behind errors remain key targets for improvement and future innovations in community pharmacies.

### Study limitations and strengths

The national DE register is based on voluntary self-reporting and is primarily intended for community pharmacies’ internal quality management; it is not mainly used for collecting research data. Although the AFP has published reporting guidelines for community pharmacies to support DE reporting and classification in their system, there was inconsistency in reporting and classifying DEs. Thus, the data was systematically preprocessed to ensure consistency throughout the data and to strengthen the study’s validity.

The present study does not describe the actual DE incidence in Finland. This is mainly due to under-reporting, which is common in the use of voluntary self-reporting systems of medication errors and other adverse events in health- and social care, although reporting systems are a cost-effective and feasible method for long-term systems-based risk management [28, 54]. Another reason for under-reporting is that not all Finnish community pharmacies used the AFP’s DE reporting system but some other method for mandatory documentation of DEs.

Our study indicated some challenges in categorising certain types of DEs. This demonstrates the importance of DE taxonomies used in DE reporting systems as facilitators of feasible and comprehensive reporting. Structured classification systems may miss even some crucial aspects of the practice by excluding these aspects from the risk evaluation and development of safer processes. In our study, we found that the DE reporting system by the AFP was missing categories for errors related to medication counselling and dispensing over-the-counter medicines. Also, more detailed categories for errors related to multidose dispensing would be needed.

The year 2020, included in this study, was the first year of the global COVID-19 pandemic. In Finland, community pharmacies managed to operate exceptionally well in the exceptional circumstances due to COVID-19; for example, laboratory-confirmed COVID-19 infections were detected in only 4% of community pharmacies during 2020, and no internal chains of infection occurred in any of them [51]. Also, closures of community pharmacies were almost wholly avoided. However, uncertainty and the need to quickly implement new agile operation models have possibly caused stress in community pharmacies, which may have affected DE reporting in community pharmacies despite the obligation of the DE reporting.

### Future perspectives

After this study, Finnish community pharmacies have extended their reporting scope from DEs in prescription dispensing to all medication errors in outpatient care. The AFP, with the Finnish Centre for Client and Patient Safety, launched the National Medication Safety Programme for Community Pharmacies in Finland to support this strategic change by 2026 [62]. As part of this long-term programme, in 2021, a more comprehensive patient safety reporting and learning system (HaiPro) that has been widely used in Finnish health- and social care since 2007 replaced the AFP’s DE reporting system [63]. The HaiPro system responds to the need to develop the taxonomy for error reporting in community pharmacies, and it enables community pharmacies to report also near misses and issues with over-the-counter medicines. Using the same system in community pharmacies and other health- and social care organisations enables the sharing of incident reports between the above-mentioned organisations. For example, community pharmacists can report a prescribing error detected while dispensing to the healthcare organisation where the error occurred. This provides the healthcare organisation with new information about prevailing risks in their medication management processes. Also, community pharmacies can receive reports from health- and social care organisations, such as information on an error in a patient’s medication counselling that occurred in a community pharmacy but was detected in the health centre. In addition to using incident reports at the organisational level, community pharmacies’ incident reports can be used at the regional and national levels to develop risk management actions and procedures.

## Conclusions

The reported DEs and almost all DE types in Finnish community pharmacies decreased significantly in 2015–2020. These changes seem to be associated with digitalisation and new technologies implemented in the dispensing process in Finland, particularly, the implementation of the MVS and the additional safety feature integrated into the MVS process in 2019. The role of patients and pharmacy personnel in detecting DEs has remained central regardless of changes in dispensing practices. In the future, more in-depth research into the risks of the dispensing process and their contributing factors is needed, as well as how they can be influenced e.g. by implementing new digitalisation technologies as defences. In addition, classifications in community pharmacy medication error reporting systems should cover all crucial aspects of practice with sufficient detail, such as medication counselling, dispensing over-the-counter medicines and multidose dispensing.

### Supplementary Information

Additional file 1:  The electronic reporting form of the national dispensing error register of Finnish community pharmacies.

Additional file 2: The taxonomy of dispensing error types of Finnish community pharmacies’ national dispensing error register.

Additional file 3: Data processing before performing quantitative analysis of the study.

Additional file 4: Rates of contributing factors to dispensing errors selected by community pharmacies.

## Data Availability

The datasets analysed during the current study are not publicly available. The access to datasets needs to be separately negotiated with the organisation (the Association of Finnish Pharmacies) owning the data.

## Abbreviations

AFP: The Association of Finnish Pharmacies
DE: Dispensing error
MVS: The Medicines Verification System
