# Supplementary material for: Trends in dispensing errors reported in Finnish community pharmacies in 2015–2020: a national retrospective register-based study
Source: BMC Prim Care. 2024 May 23;25:183. doi: 10.1186/s12875-024-02428-y (PMC11118726; doi:10.1186/s12875-024-02428-y)
Supplement: Supplementary file 1 — Additional file 1: The electronic reporting form of the national dispensing error register of Finnish community pharmacies. [file 12875_2024_2428_MOESM1_ESM.pdf]

ADDITIONAL FILE 1

The electronic reporting form of the national dispensing error register of Finnish community pharmacies. Mandatory parts of the form are marked with an asterisk (*). Information used in this study is marked with bold letters.

REPORTABLE INFORMATION	REPORTING FORM OF REPORTABLE INFORMATION
The community pharmacy where the dispensing error occurred*	The name of the community pharmacy is filled in automatically
The person who completed the form*	For example, initials
Reference information	For example, a prescription number or other anonymous information, which can be used to trace error-related information from the pharmacy information system
**Prescription type***	○ Electronic prescription<br>○ Fax prescription<br>○ Hand-written prescription<br>○ Computer-generated paper prescription<br>○ Telephone prescription
**The medicine that was supposed to be dispensed***	Select from the list or select “Other medicine” and write the name, strength, dosage form and quantity/pack size of the medicine
**The medicine that was dispensed***	Select from the list or select “Other medicine” and write the name, strength, dosage form and quantity/pack size of the medicine
Was the medicine used?*	○ Yes<br>○ No<br>○ Not known
**Dispensing error type***	○ Error in preparing medication for administration<br>○ Error related to multidose dispensing<br>○ Dispensing entries made incorrectly by the pharmacy<br>○ Incorrectly recorded dosage instructions at the pharmacy<br>○ Pricing error<br>○ Wrong dosage form<br>○ Wrong generic medicine<br>○ Wrong medicine<br>○ Wrong quantity or pack size<br>○ Wrong strength<br>○ Wrong person or name<br>○ Another type of dispensing error, please specify

**Contributing factor(s) to the dispensing error**	Several options can be selected:<br>☐ Factor related to employee<br>☐ Factor related to generic substitution<br>☐ Factor related to medicine storage<br>☐ Factor related to the patient (incl. relatives of patients)<br>☐ Factor related to the situation<br>☐ Factor related to working conditions<br>☐ Similar names of the medicinal products<br>☐ Similar packaging of the medicinal products<br>☐ Unclear prescription<br>☐ Another factor<br><br>Please specify further clarification of contributing factor(s) if applicable
**The individual who detected the dispensing error***	○ Nurse<br>○ Patient<br>○ Pharmacy personnel (incl. pharmacists and pharmacy technicians)<br>○ Physician<br>○ Relative of the patient<br>○ Other, please specify
**Date of dispensing***	Select from the calendar
**Date when the dispensing error was detected***	Select from the calendar
Did the dispensing error cause patient harm?*	○ Yes, adverse effect or another symptom, please specify<br>○ Yes, missing response or effect, please specify<br>○ No<br>○ Not known
Treatment actions for the patient	Several options can be selected:<br>☐ A physician was contacted by the pharmacy<br>☐ The patient was directed to the physician's appointment, or the patient had already visited a physician's appointment<br>☐ The patient was hospitalised<br>☐ Other treatment action, please specify
Corrective actions for the dispensing error	Several options can be selected:<br>☐ The right medicine, strength or quantity/pack size dispensed to the patient<br>☐ The dosage instructions of the medicine corrected<br>☐ The difference in the price of the medicines was refunded to the patient<br>☐ Necessary corrections made to the electronic stock control system<br>☐ Necessary corrections informed to the Social Insurance Institution (Kela) of Finland<br>☐ Other corrective action, please specify

**Further clarification of the dispensing error event**	Please specify if applicable
Actions and changes in working procedures to prevent dispensing errors in the pharmacy	Please specify if applicable
