# Supplementary material for: Trends in dispensing errors reported in Finnish community pharmacies in 2015–2020: a national retrospective register-based study
Source: BMC Prim Care. 2024 May 23;25:183. doi: 10.1186/s12875-024-02428-y (PMC11118726; doi:10.1186/s12875-024-02428-y)
Supplement: Supplementary file 2 — Additional file 2: The taxonomy of dispensing error types of Finnish community pharmacies’ national dispensing error register. [file 12875_2024_2428_MOESM2_ESM.pdf]

ADDITIONAL FILE 2

The taxonomy of dispensing error types of Finnish community pharmacies' national dispensing error register.

DISPENSING ERROR TYPE	DEFINITION
**Wrong medicine**	Dispensed wrong medicine (wrong medicinal substance)
**Wrong strength**	Dispensed wrong strength from the right medicine
**Wrong dosage form**	Dispensed wrong dosage form (e.g. prolonged-release capsule – tablet)
**Wrong quantity or pack size**	Dispensed the wrong quantity or pack size from the right medicine
**Wrong generic medicine**	Dispensed the right medicinal substance from the wrong pharmaceutical manufacturer
**Wrong person or name**	Dispensed another patient's medicine, or the wrong patient's name entered on the dispensed medication package
**Incorrectly recorded dosage instructions at the pharmacy**	Dispensed the right medicine with the wrong recorded dosage instructions
**Error in preparing medicine for administration**	E.g. dosage instructions labels were attached to the wrong medication package or not enough water was added to the antibiotic suspension
**Pricing error**	The right medicine was dispensed according to the prescription, but the wrong medicine was selected from the pricing database
**Dispensing entries made incorrectly by the pharmacy**	Dispensing entries made incorrectly to the prescription (e.g. remaining or total quantity on prescription)
**Error related to multidose dispensing**	A dispensing error occurred at any stage in the multidose dispensing process
**Another type of dispensing error**	Another dispensing error type than listed above
