# Supplementary material for: Trends in dispensing errors reported in Finnish community pharmacies in 2015–2020: a national retrospective register-based study
Source: BMC Prim Care. 2024 May 23;25:183. doi: 10.1186/s12875-024-02428-y (PMC11118726; doi:10.1186/s12875-024-02428-y)
Supplement: Supplementary file 3 — Additional file 3: Data processing before performing quantitative analysis of the study. [file 12875_2024_2428_MOESM3_ESM.pdf]

ADDITIONAL FILE 3

Data processing before performing quantitative analysis of the study.

<table>
<tr>
<td>Deleted cases from the data</td>
<td>

Duplicate cases
Cases that did not fulfil the definition of a dispensing error

Errors that were detected and corrected before the medicine or multidose dispensed medicine was dispensed from a community pharmacy, such as

The wrong medication package was collected, but it was changed to the right medication package before dispensing to the patient

Prescribing errors and other errors that occurred elsewhere in health and social care, such as

The patient was given the wrong person's medication treatment instruction
The change in the multidose dispensing order has not been informed to the community pharmacy, and consequently, the order was processed according to the earlier instruction

Errors in selling over-the-counter medicines
Errors related to direct compensation of medicines granted by the Social Insurance Institution of Finland (Kela)

The community pharmacy gave the wrong amount of direct compensation for the dispensed product
Direct compensation granted by the Kela was given too early, i.e., dispense intervals specified by the Kela were not considered
More than 3-month worth of the medicine was dispensed from the community pharmacy

Deliberate deviations from prescriptions made due to pharmacists' consideration, such as

The medicine has been exchanged for a non-generic one due to a medicine shortage of the prescribed one (e.g. exchange from a hormonal contraceptive to an available manufacturer’s preparation (same medicinal substance and strength) or exchange from a cream to a gel)

</td>
</tr>
<tr>
<td>Added cases to the data</td>
<td>

Cases related to more than one medicine dispensed were recorded as separate dispensing errors according to the medicine, such as

The dosing instruction labels of dispensed medicines were mixed so a separate dispensing error was recorded for each medicine

Cases that included more than one dispensing error type were recorded as separate dispensing errors according to the error types, such as

Both the wrong strength and pack size of the medicine were dispensed to the patient so two separate dispensing errors were recorded

Cases that had recurred more than once per patient were recorded as separate dispensing errors according to the number of recurrences, such as

The wrong strength of medicine was dispensed to the patient twice so two separate dispensing errors were recorded

</td>
</tr>
</table>

**Corrected cases from the data**	• Dispensing error types that were incorrectly categorised by community pharmacies, such as<br>○ The type of dispensing error was categorised as the wrong strength by the community pharmacy even though the correct type of error was the wrong medicine<br>• "Individuals who detected the dispensing errors" that were incorrectly categorised by community pharmacies, such as<br>○ Animal owners that were categorised under several different categories (patient, relative of patient, nurse, other) by community pharmacies were systematically classified into the "Other" category
