# Supplementary material for: Trends in dispensing errors reported in Finnish community pharmacies in 2015–2020: a national retrospective register-based study
Source: BMC Prim Care. 2024 May 23;25:183. doi: 10.1186/s12875-024-02428-y (PMC11118726; doi:10.1186/s12875-024-02428-y)
Supplement: Supplementary file 4 — Additional file 4: Rates of contributing factors to dispensing errors selected by community pharmacies. [file 12875_2024_2428_MOESM4_ESM.pdf]

ADDITIONAL FILE 4

Rates of contributing factors to dispensing errors selected by community pharmacies. Rates = annual numbers of contributing factors (n) and annual numbers of contributing factors proportioned to the number of dispensing error reports per year (%).

Contributing factors	2015	2016	2017	2018	2019	2020	TOTAL
	n<br>*%*	*n*<br>*%*	*n*<br>*%*	*n*<br>*%*	*n*<br>*%*	*n*<br>*%*	*n*<br>*%*
Factor related to employee	1423<br>36.4	1400<br>36.9	1415<br>38.2	1219<br>34.1	842<br>34.5	743<br>35.1	7042<br>36.0
Similar packaging of the medicinal products	1091<br>27.9	1084<br>28.6	1080<br>29.1	1058<br>29.6	530<br>21.7	297<br>14.0	5140<br>26.3
Similar names of the medicinal products	811<br>20.7	792<br>20.9	775<br>20.9	782<br>21.9	535<br>21.9	452<br>21.4	4147<br>21.2
Factor related to the situation	620<br>15.8	514<br>13.5	590<br>15.9	618<br>17.3	494<br>20.3	395<br>18.7	3231<br>16.5
Factor related to generic substitution	443<br>11.3	457<br>12.0	522<br>14.1	588<br>16.4	457<br>18.7	508<br>24.0	2975<br>15.2
Factor related to medicine storage	247<br>6.3	280<br>7.4	314<br>8.5	410<br>11.5	216<br>8.9	144<br>6.8	1611<br>8.2
Factor related to working conditions	270<br>6.9	264<br>7.0	254<br>6.9	277<br>7.7	202<br>8.3	172<br>8.1	1439<br>7.4
Factor related to the patient (incl. relatives of patients)	122<br>3.1	105<br>2.8	126<br>3.4	133<br>3.7	115<br>4.7	96<br>4.5	697<br>3.6
Unclear prescription	71<br>1.8	78<br>2.1	53<br>1.4	54<br>1.5	48<br>2.0	59<br>2.8	363<br>1.9
Other contributing factors	364<br>9.3	364<br>9.6	355<br>9.6	366<br>10.2	314<br>12.9	304<br>14.4	2067<br>10.6
**Total of all contributing factors** *(n)*	5462	5338	5484	5505	3753	3170	28712
**DE reports per year** *(n)*	3913	3795	3708	3578	2439	2117	19550
**Mean of contributing factors per DE report** *(n)*	1.4	1.4	1.5	1.5	1.5	1.5	1.5
**DE reports in which contributing factors had not been defined**	173<br>4.4	119<br>3.1	124<br>3.3	125<br>3.5	96<br>3.9	106<br>5.0	743<br>3.8
